# Impact of telemedical management on hospitalization and mortality in heart failure patients with diabetes: a post-hoc subgroup analysis of the TIM-HF2 trial

**DOI:** 10.1186/s12933-024-02285-0

**Published:** 2024-06-12

**Authors:** Friedrich Koehler, Johanna Koehler, Peter Bramlage, Eik Vettorazzi, Karl Wegscheider, Susanne Lezius, Sebastian Spethmann, Roman Iakoubov, Anjaly Vijayan, Sebastian Winkler, Christoph Melzer, Katharina Schütt, Cécile Dessapt-Baradez, W.Dieter Paar, Kerstin Koehler, Dirk Müller-Wieland

**Affiliations:** 1 Centre for Cardiovascular Telemedicine, Deutsches Herzzentrum der Charité (DHZC), Charitéplatz 1, 10117 Berlin, Germany; 2 Charité - Universitätsmedizin Berlin, Corporate member of Freie Universität Berlin and Humboldt-Universität zu Berlin, Berlin, Germany; 3 German Centre for Cardiovascular Research (DZHK), Partner Site, Berlin, Germany; 4 Department of Internal Medicine II, School of Medicine, University Hospital Rechts der Isar, Technical University of Munich, Munich, Germany; 5 Institute for Pharmacology and Preventive Medicine, Cloppenburg, Germany; 6 Institute of Medical Biometry and Epidemiology, Medical Center Hamburg-Eppendorf (UKE), Hamburg, Germany; 7 Department of Cardiology, Angiology and Intensive Care Medicine, Deutsches Herzzentrum der Charité, Charitéplatz 1, 10117 Berlin, Germany; 8 Clinic for Internal Medicine and Cardiology, BG Klinikum Unfallkrankenhaus Berlin, Berlin, Germany; 9 Department of Internal Medicine I, RWTH Aachen University Hospital, Aachen, Germany; 10 Sanofi, Reading, UK; 11 Sanofi-Aventis Deutschland GmbH, Berlin, Germany

**Keywords:** Diabetes, Heart failure, Remote patient management, Telehealth, Telemedicine

## Abstract

**Background:**

The TIM-HF2 study demonstrated that remote patient management (RPM) in a well-defined heart failure (HF) population reduced the percentage of days lost due to unplanned cardiovascular hospital admissions or all-cause death during 1-year follow-up (hazard ratio 0.80) and all-cause mortality alone (HR 0.70). Higher rates of hospital admissions and mortality have been reported in HF patients with diabetes compared with HF patients without diabetes. Therefore, in a post-hoc analysis of the TIM-HF2 study, we investigated the efficacy of RPM in HF patients with diabetes.

**Methods:**

TIM-HF2 study was a randomized, controlled, unmasked (concealed randomization), multicentre trial, performed in Germany between August 2013 and May 2018. HF-Patients in NYHA class II/III who had a HF-related hospital admission within the previous 12 months, irrespective of left ventricular ejection fraction, and were randomized to usual care with or without added RPM and followed for 1 year. The primary endpoint was days lost due to unplanned cardiovascular hospitalization or due to death of any cause. This post-hoc analysis included 707 HF patients with diabetes.

**Results:**

In HF patients with diabetes, RPM reduced the percentage of days lost due to cardiovascular hospitalization or death compared with usual care (HR 0.66, 95% CI 0.48–0.90), and the rate of all-cause mortality alone (HR 0.52, 95% CI 0.32–0.85). RPM was also associated with an improvement in quality of life (mean difference in change in global score of Minnesota Living with Heart Failure Questionnaire score (MLHFQ): − 3.4, 95% CI − 6.2 to − 0.6).

**Conclusion:**

These results support the use of RPM in HF patients with diabetes.

**Clinical trial registration:**

ClinicalTrials.gov NCT01878630.

## Introduction

Telemedical management of heart failure (HF) patients is a specialized digital care approach aimed at reducing adverse outcomes such as HF hospitalization and death [1]. The Telemedical Interventional Management in Heart Failure II (TIM-HF2) study (NCT01878630) demonstrated that remote patient management (RPM), when used in a well-defined HF population, significantly reduced the percentage of days lost due to unplanned cardiovascular hospital admissions or all-cause death during 1-year follow-up by 20% ( 95%CI: 0.65-1.00) and all-cause mortality alone by 30% ( 95% CI: 0.50 − 0.96) compared to usual care [2].

Even though higher hospitalization and mortality rates in HF-patients with diabetes have been reported compared with patients without diabetes [3–5], the most recent (2023) European Society of Cardiology (ESC) guidelines on the management of cardiovascular disease in patients with diabetes lack a clear position on the benefits of RPM in these patients [1]. Recent guidelines by the American Diabetes Association (ADA) [6] acknowledge the emerging role of telehealth, including remote patient monitoring; however, they focus primarily on blood glucose control. Due to the lack of evidence, there are no specific recommendations for patients with diabetes and HF.

Using the data from the TIM-HF2 study, we investigated whether RPM was an effective tool to manage this high-risk patient population.

## Research design and methods

### TIM-HF2 study design

Details of the TIM-HF2 trial have been reported previously [2]. Briefly, TIM-HF2 was a, randomized, controlled, unmasked (with concealed randomization), multicentre trial performed in Germany between August 2013 and May 2018. Participants were enrolled in 200 study centres allocated in hospitals, in cardiology practices and general practitioner practices. The study complied with good clinical practice in accordance with the Declaration of Helsinki and German law, and was approved by the appropriate ethics committees. Patients provided written informed consent.

The TIM-HF2 cohort comprised HF-patients with New York Heart Association (NYHA) class II/III HF who had a HF-related hospital admission within the previous 12 months, and had left ventricular ejection fraction (LVEF) ≤ 45% or if > 45% were receiving diuretics [2, 7]. Patients were excluded if they had major depression, were on haemodialysis, had been hospitalized within the previous 7 days, had a LV assist device, had undergone coronary revascularization or cardiac resynchronization therapy within the previous 28 days, or were scheduled to undergo either of these procedures or aortic or mitral valve procedures within the next 3 months.

### Randomisation and masking

Patients were randomized using a secure web-based system to receive usual care with or without added RPM, and then followed for one year. Pocock’s minimization algorithm with 10% residual randomness was used to ensure a balance of important clinical covariates between the groups. Randomization was concealed, but participants and investigators were not blinded as to group assignment. Clinical endpoints were adjudicated by an endpoint committee blinded to study group assignment.

### Remote patient management

Details of the RPM intervention have been published previously [2, 7]. RPM consisted of a daily transmission of vital parameters from the home of the patient to a telemedical centre (TMC) serving 24 h/7 days a week, remote patient education provided by HF nurses during a scheduled monthly telephone interview, and close cooperation between the primary treating physicians and the staff of the telemedical centre. Telemonitoring was performed using a non-invasive multiparameter home-monitoring system with a three-channel ECG device (able to collect 2-minute or streamed measurements), a blood pressure measuring device, and weighting scales as previously described. Transmitted parameters included bodyweight, systolic and diastolic blood pressure, heart rate, ECG, peripheral capillary oxygen saturation, and a self-rated health status (on a scale from 1 to 5).

The telemonitoring system used a wireless system and digital tablet to transmit data from the patient’s home to the TMC via the mobile phone network (secured using a virtual private network tunnel). The TMC provided 24-hour physician-led patient management. CE-marked telemedical analysis software incorporating algorithms guided patient management, enabling TMC physicians to act promptly as necessary. Transmitted vital parameters, reported symptom status and medications for every patient were evaluated by TMC staff every day. When necessary, medication changes, unplanned ambulatory physician visits, or hospitalizations were initiated by a TMC physician. The telemonitoring system allowed direct communication between TMC staff and patients and the patients’ GPs and cardiologists.

Patients were given training on the telemonitoring system, and were provided with education about heart failure during a monthly structured telephone interview with a HF-nurse. Patients were given mobile phones to contact TMC staff directly in case of emergency.

All patients were seen by their treating cardiologist at baseline and at 1 year. At 3, 6, and 9 months they were seen by their GP or cardiologist. Data including vital signs, bodyweight and the occurrence of hospital admission were recorded at these visits. Data on hospital admissions were confirmed by cross-checking information provided by the investigators with data from patients’ health insurance companies.

### Post hoc analysis of patients with diabetes

Patients with comorbid diabetes were identified post-hoc using clinical assessment results and the presence of antidiabetic drug prescriptions (oral antidiabetic drugs and/or insulin) at the baseline visit. Diabetes-specific laboratory values (e.g., HbA1c, fasting plasma glucose) were not obtained and a diabetes-specific intervention was not incorporated into RPM.

The primary endpoint for the overall study as well as for this post hoc analysis was “days lost due to unplanned cardiovascular hospitalization or death from any cause” during the 1-year follow-up. Key secondary endpoints were all-cause mortality, cardiovascular mortality, and quality of life as defined by the Minnesota Living with Heart Failure Questionnaire (MLHFQ).

### Statistical analysis

The proportion of follow-up time lost to death or unplanned cardiovascular hospitalization was defined as the number of days lost divided by the intended follow-up. For patients who died, the number of days lost between the date of death and the end date of intended follow-up plus any days spent in hospital for cardiovascular reasons were counted. For patients who completed follow-up or who withdrew prematurely, the fraction of follow-up was defined as the number of days lost due to cardiovascular hospitalization divided by the follow-up time achieved.

Baseline characteristics were summarized as the number of patients (%) for categorical variables and as the mean (SD) for continuous variables. For the primary outcome, weighted averages of the percentage of days lost were compared between the groups using a permutation test based on 2000 randomly drawn permutations. The two-sided p-value was calculated as the fraction of permutations which had a test statistic absolute value at least as large as the observed test statistic (with a mid-p correction applied in case of equality). Follow-up time was weighted using weighted arithmetic means and presented as an annualized average.

Survival analyses were performed on a time-to-event basis. Cumulative incidence curves for all-cause and cardiovascular mortality were constructed using the Kaplan-Meier method. The differences between curves were examined using log-rank statistics (all-cause mortality) or the Gray’s test (cardiovascular mortality). Cox-proportional hazards regression models were used to estimate (cause-specific) hazard ratios (HRs).

## Results

Among 1538 patients included in the TIM-HF2 cohort between 13 August 2013 and 12 May 2017. Among them, 707 patients (46%) were diagnosed with comorbid diabetes and 831 patients (54%) did not have diabetes.

Baseline characteristics were generally similar in the diabetes and non-diabetes groups; however, patients with diabetes had higher rates of functional class NYHA III (57% vs. 39%), hyperlipidaemia (64% vs. 46%), previous coronary disease/myocardial infarction (50% vs. 32%), coronary revascularization (41% vs. 32%), and coronary artery bypass surgery (24% vs. 13%) (Table 1).

**Table 1:** Patient characteristics at baseline

Characteristic	Diabetes				No diabetes
	Overall (*N* = 707)	Usual Care (*N* = 359)	RPM (*N* = 348)	p-value*	Overall (*N* = 831)
Male gender	502 (71%)	258 (72%)	244 (70%)	0.62	568 (68%)
Age (years)	71.1 (9.2)	71.1 (9.0)	71.0 (9.4)	0.91	69.7 (11.5)
BMI (kg/m²)	31.6 (6.3)	31.9 (6.2)	31.3 (6.5)	0.17	28.1 (5.6)
Systolic BP (mm Hg)	126.8 (19.3)	127.2 (19.9)	126.5 (18.7)	0.63	124.6 (19.3)
Pulse (bpm)	73.6 (13.7)	73.4 (13.9)	73.8 (13.6)	0.71	71.1 (14.1)
Living alone	195 (28%)	97 (27%)	98 (28%)	0.74	240 (29%)
*NYHA class*				0.54	
I	1 (0.1%)	1 (0.3%)	0 (0%)		10 (1.2%)
II	303 (43%)	148 (41%)	155 (45%)		493 (59%)
III	400 (57%)	209 (58%)	191 (55%)		326 (39%)
IV	3 (0.4%)	1 (0.3%)	2 (0.6%)		2 (0.2%)
LVEF	41.7 (12.8)	41.3 (12.5)	42.0 (13.1)	0.45	40.3 (13.8)
LVEF				0.18	
HFrEF	355 (50%)	183 (51%)	172 (49%)		463 (56%)
HFmrEF	118 (17%)	67 (19%)	51 (15%)		106 (13%)
HFpEF	234 (33%)	109 (30%)	125 (36%)		262 (32%)
*Kidney function*					
Serum creatinine (µmol/L)	127.9 (48.4)	130.5 (49.2)	125.1 (47.3)	0.14	114.7 (43.9)
eGFR (mL/min/1.73m^2^)	57.6 (23.7)	57.2 (23.8)	58.1 (23.6)	0.64	62.9 (22.2)
Primary cause of chronic heart failure				0.11	
Coronary Disease/MI	357 (50%)	190 (53%)	167 (48%)		267 (32%)
Hypertension	135 (19%)	74 (21%)	61 (18%)		139 (17%)
Other	101 (14%)	42 (12%)	59 (17%)		192 (23%)
Cardiomyopathy	114 (16%)	53 (15%)	61 (18%)		233 (28%)
*Antidiabetic medications*					
Insulin use	339 (48%)	169 (47%)	170 (49%)	0.65	0 (0.0%)
Oral hypoglycaemic drugs	388 (55%)	183 (51%)	205 (59%)	0.035	0 (0.0%)
Smoker	47 (6.6%)	20 (5.6%)	27 (7.8%)	0.39	87 (10%)
COPD	155 (22%)	80 (22%)	75 (22%)	0.86	119 (14%)
Hyperlipidaemia	454 / 707 (64%)	236 / 359 (66%)	218 / 348 (63%)	0.43	379 / 830 (46%)
Coronary revascularization	292 / 706 (41%)	162 / 359 (45%)	130 / 347 (37%)	0.039	268 / 831 (32%)
Coronary artery bypass surgery	172 (24%)	91 (25%)	81 (23%)	0.54	107 (13%)
ICD implanted	212 (30%)	103 (29%)	109 (31%)	0.46	244 (29%)
CRT device	125 (18%)	61 (17%)	64 (18%)	0.69	115 (14%)

* Fisher’s exact test for the comparison of usual vs. remote care; Welch Two Sample t-test. Continuous variables are presented as mean (standard deviation), discrete as absolute numbers (percent)

BMI, body mass index; BP, blood pressure; bpm, beats per minute; COPD, chronic obstructive pulmonary disease; CRT, cardiac resynchronization therapy; eGFR, estimated glomerular filtration rate; HFmrEF, heart failure with mildly reduced ejection fraction; HFpEF, heart failure with preserved ejection fraction; HFrEF, heart failure with reduced ejection fraction; ICD, implantable cardioverter defibrillator; LVEF, left ventricular ejection fraction; MI, myocardial infarction; NYHA, New York Heart Association; RPM, remote patient management; SD, standard deviation

**Fig. 1:**
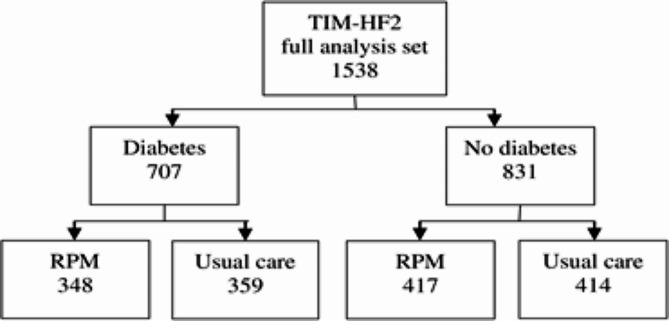
Post hoc analysis profile. RPM, remote patient management

Among patients with diabetes, the baseline characteristics of the RPM and usual-care groups were similar, apart from a lower rate of oral hypoglycaemic drug use (51% vs. 59%, *p* = 0.035) and higher rate of coronary revascularization (45% vs. 37%, *p* = 0.039) in the RPM group (Table 1). Among diabetes patients, 348 received RPM in addition to usual care and 359 received usual care alone (Fig. 1). In the usual-care group the rate of the primary endpoint (days lost due to unplanned cardiovascular hospitalization or death from any cause) was 44% (157/359) in patients with diabetes and 32% (133/414) in patients without diabetes respectively, whereas in the RPM group the primary endpoint was met by 35% (122/348) diabetes patients and 34% (143/417) non-diabetic patients respectively. In summary, RPM significantly reduced the percentage of days lost due to cardiovascular hospitalization or death (primary endpoint) compared to usual care during the 1-year follow-up period in diabetes patients only (weighted average 4.57% vs. 7.71%; ratio 0.66; 95% CI 0.48, 0.90; *p* = 0.009) (Table 2). The average number of days lost per year was 16.7 in the RPM group versus 28.1 days in the usual-care group.

**Table 2:** Primary and key secondary outcomes in patients with diabetes

	RPM		Usual Care		RPM versus Usual Care	
	Number of patients with events	Weighted average (95% CI)	Number of patients with events	Weighted average (95% CI)	Ratio (95% CI)	p value
Percentage of days lost due to unplanned cardiovascular hospitalization or death of any cause	122/348 (35%)	4.57% (3.22, 5.93)	157/359 (44%)	7.71% (5.88, 9.54)	0.66* (0.48, 0.90)	0.009
Number of days lost per year		16.7 (11.8, 21.6)		28.1 (21.5, 34.8)		
All-cause mortality	25/348 (7%)	7.11 (4.81, 10.52)	49/359 (14%)	13.58 (10.26, 17.97)	0.52^†^ (0.32, 0.85)	0.008
Cardiovascular mortality	17/348 (5%)	4.84 (3.01, 7.78)	30/359 (8%)	8.32 (5.81¸11.89)	0.58^†^ (0.32, 1.05)	0.073

CI, confidence interval; RPM, remote patient management; * ratio of the weighted average; †hazard ratio

**Fig. 2:**
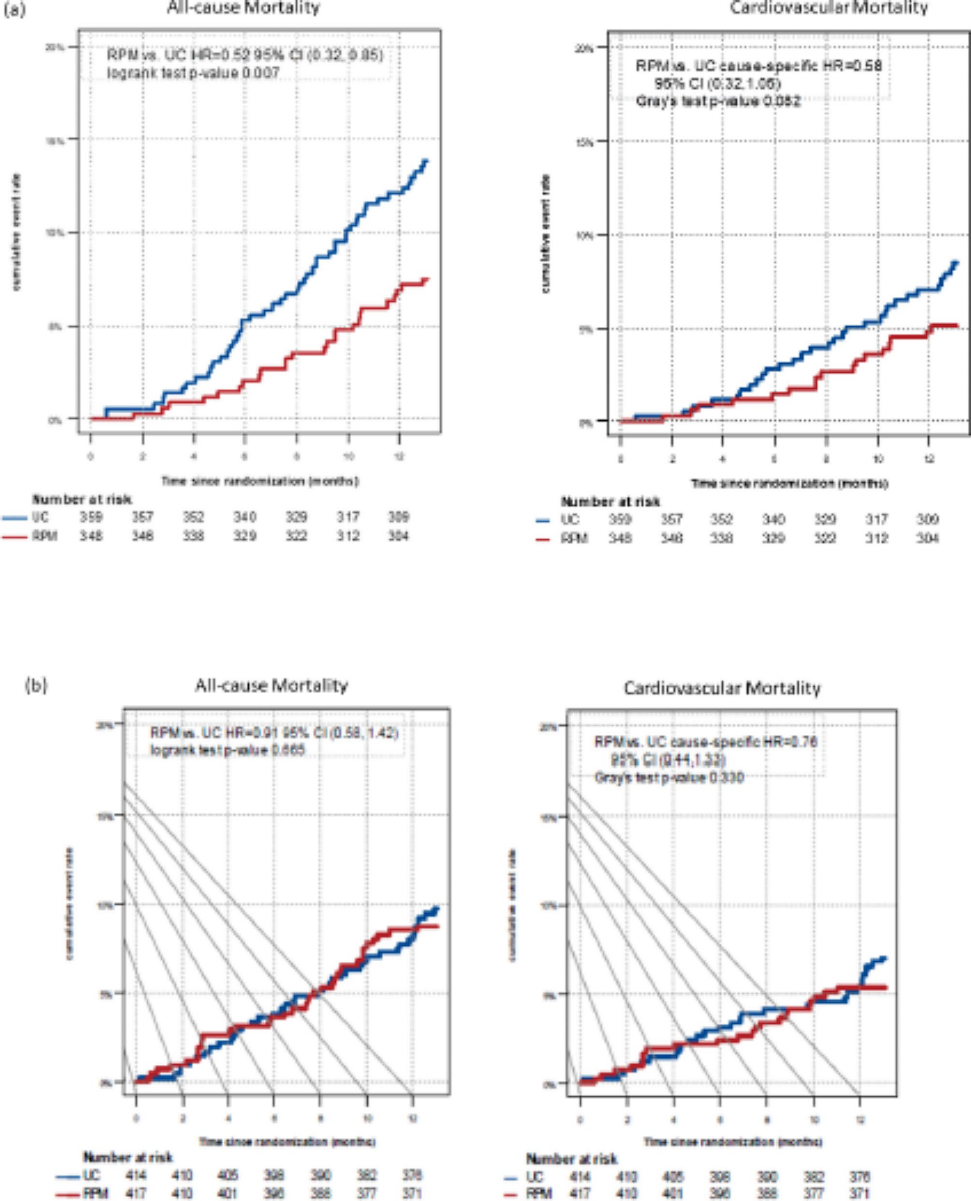
Kaplan-Meier cumulative event curve for all-cause (**left**) and cardiovascular (**right**) mortality for (**a**) patients with diabetes and (**b**) patients without diabetes. RPM, remote patient management; UC, usual care

Among patients with diabetes, the all-cause mortality rate was reduced in the RPM group (weighted average 7.11% vs. 13.58%), with a hazard ratio of 0.52 (95% CI 0.32, 0.85; *p* = 0.008); however, the risk reduction for cardiovascular mortality did not reach statistical significance (4.84% vs. 8.32%; ratio 0.58; 95% CI 0.32, 1.05; *p* = 0.073; Table 2; Fig. 2a). In non-diabetic patients, no significant difference in all-cause mortality between groups was seen (Fig. 2b**)**.

Among patients with diabetes, RPM improved quality of life compared to usual care, as indicated by an estimated mean difference in the change in MLHFQ global score of − 3.4 (95% CI − 6.2, − 0.6) (Table 3). In the TIMI-HF2 subgroup of patients without diabetes, RPM did not improve quality of life compared with usual care (mean difference in change in MLHFQ global score 0.7, 95% CI − 1.8, 3.3).

**Table 3:** Quality of life in patients with diabetes according to type of care received: change from baseline to 365 days

	*N*	RPM Est. mean change (95% CI)	*N*	Usual Care Est. mean change (95% CI)	RPM vs. Usual Care Est. mean difference (95% CI)
MLHFQ Global Score	292	− 2.1 (− 4.1, − 0.1)	276	1.3 (− 0.7, 3.4)	− 3.4 (− 6.2, − 0.6)
MLHFQ Physical Function	292	− 2.4 (− 4.5, − 0.3)	276	1.3 (− 0.8, 3.4)	− 3.7 (− 6.6, − 0.8)
MLHFQ Emotional	292	− 2.8 (− 4.9, − 0.8)	276	0.5 (− 1.6, 2.6)	− 3.3 (− 6.3, − 0.4)

MLHFQ, Minnesota Living with Heart Failure Questionnaire; RPM, remote patient management

## Discussion

Here, we present evidence that in patients with HF and diabetes, RPM was associated with reduced all-cause mortality compared with usual care. This survival benefit in patients with diabetes was observed either alone or in combination with a reduction of the percentage of days lost due to unplanned cardiovascular hospital admissions due to HF. Our results also indicate that RPM improved quality of life among patients with HF and diabetes compared with usual care.

Diabetes was recently reported to be an independent risk factor for first HF hospitalization (HR 1.437; 95% CI 1.078, 1.917), along with other covariables, such as age (HR 1.026), N-terminal pro-B-type natriuretic peptide (HR 1.275), myocardial infarction (HR 1.560), and chronic obstructive pulmonary disease (HR 1.742) [8]. These findings support our data which shows that the rate of days lost due to unplanned cardiovascular hospitalization or death from any cause was 44% in patients with diabetes compared to 32% in patients without diabetes in the usual-care group. Furthermore, diabetic patients in the TIM-HF2 trial had an increased prevalence of coronary disease/myocardial infarction (50% vs. 32%) and COPD (22% vs. 14%) compared to non-diabetic patients, highlighting the importance of using diabetes as a clinical indicator to characterize a high-risk HF-patient population that may benefit from RPM.

Several systematic reviews have concluded that telemonitoring can reduce the risk of all-cause mortality or HF-related hospitalizations among patients with HF [9–11]. However, some studies have reported different results, possibly due to a wide variation in the approaches used [12, 13]. Telemonitoring of HF patients often focuses on sensor technology, rather than the time spent on direct patients monitoring or actions implemented by the available physician and/or HF nurse support. However, improved monitoring and prompt implementation of appropriate treatment changes represent the essence of telemedicine [13], with the frequency or intensity of monitoring and the availability of associated medical support reported to play an important role [13, 14]. Several studies that did not observe remote monitoring benefits evaluated only alerts during working hours and/or notified nurses to prompt patients to contact their doctor [15, 16], resulting in minimal integration with physician care and the absence of rapid changes in treatment.

In contrast, the intensive interventional approach used in the TIM-HF2 study allowed patient data to be immediately transmitted to the telemedical centre, guiding patient care in real-time and facilitating individualized treatment in a timely manner. The main goal of the RPM system was to detect early evidence of cardiac decompensation and to promptly initiate appropriate treatment. Therefore, timely intervention resulted in an early adjustment of evidence-based recommended medication, improving patients’ hemodynamic status.

The primary analysis of the total TIM-HF2 cohort found that this structured RPM intervention significantly reduced the primary endpoint (percentage of days lost to unplanned cardiovascular hospitalizations or all-cause mortality) compared with usual care (4.88% vs. 6.64%, *p* = 0.046), as well as the all-cause death rate (7.86 vs. 11.34 per 100 person-years, *p* = 0.028) [2]. The results of our analysis of the subgroup of diabetic patients followed a pattern similar to that observed in the overall TIM-HF2 analysis [2]; however, the effect was numerically greater in the diabetes subgroup for the primary endpoint, with a ratio of 0.66 (95% CI 0.48, 0.90) for RPM versus usual care among diabetic patients compared with a ratio of 0.80 (95% CI 0.65, 1.00) for the overall study population. The effect was also numerically greater in the diabetes subgroup for all-cause mortality (hazard ratio 0.52 [95% CI 0.32, 0.85] vs. 0.70 [95% CI 0.50, 0.96]). These differences should be interpreted with caution given the overlapping CIs, but are nonetheless interesting. Additional studies of RPM evaluating similar outcomes in patients with HF and comorbid diabetes would be of interest to confirm the findings.

Previous studies of telemedicine interventions in patients with both HF and diabetes have used different interventions and evaluated different outcomes, with varying results. A study in the USA evaluating a 3-month mobile health intervention aimed at improving physical activity and medication adherence in patients with both HF and diabetes found that it improved daily step count and health-related quality of life, but not medication adherence, compared with usual care [17]. Another US study found that a telehealth targeted medication review programme was able to successfully identify patients with type 2 diabetes and either HF or atherosclerotic cardiovascular disease who might benefit from receiving evidence-based medications, and notify their healthcare provider; however, only 6% of patients received the recommended medications within 4 months of the intervention [18]. Elsewhere, a sub analysis of the TELEREH-HF trial in Poland found that a 9-week hybrid telerehabilitation programme improved cardiopulmonary parameters in patients with HF with reduced ejection fraction who were not diabetic, but not in those with diabetes [19].

Some telemedicine interventions may improve quality of life in patients with both HF and diabetes, as seen in the US study referred to above that evaluated a mobile health intervention to improve physical activity [17]. The current study found an improvement in quality of life for patients with HF and diabetes who received the RPM intervention compared with usual care, a finding that was not observed in the overall TIM-HF2 population [2]. Patients with major depression were excluded from the study, and the baseline quality of life was reasonably good, which may explain why a significant improvement was not detected at study end for the overall population. The reason for the difference seen in the diabetic subgroup is not clear.

The analysis had several limitations. While this dataset was collected in a clinical study setting with a high completeness and internal validity, the group assignment into patients with or without diabetes was performed post-hoc and based on antidiabetic medication provided and a clinical diagnosis of diabetes at baseline. Therefore, only limited characterization of diabetes (e.g. diabetes duration, glucose control, anti-diabetic medication) was possible. The study groups were based on diabetes status at baseline and did not account for patients diagnosed with diabetes during the study period. Interventions to improve blood glucose control were not mandated by the study protocol. Patients were enrolled between 2013 and 2017 and the results of this analysis reflected the status of RPM technology and the status of optimal medical therapy during this period, particularly the status of HF therapy with SGLT-2 inhibitors were missing. Telemedical services and sensor technology have evolved in recent years, which could affect the generalizability of the data to current practice. Finally, the study was conducted in a single country (Germany) and the results may influenced when HF patients with diabetes were treated with RPM in different healthcare systems.

In conclusion, our results indicate that a structured holistic RPM intervention reduced the time spent in hospital for unplanned cardiovascular reasons and improved the survival rate of HF patients with comorbid diabetes, suggesting that RPM could have a role in managing this vulnerable patient group. Future RPM systems could incorporate additional diabetes-specific treatment targets to increase the benefit of RPM in this subset of the HF population.

## Limitation

As the study protocol was designed according to ESC guidelines for management of chronic heart failure the follow-up visits were scheduled every three months in patients with recent hospitalisation for heart failure. However, the German national guidelines for treatment of chronic heart failure do not provide specific intervals for scheduling outpatient visits after being hospitalized for chronic heart failure [20]. It is to be assumed, that the three-month follow-up in the usual care group performed in this study by cardiologists and general practitioners may be considered as an advanced care compared to current clinical practice. Therefore, there is a bias in the usual care group, which was given advanced care according to the study protocol and therefore better compliance and treatment cannot be excluded.

## Data Availability

No datasets were generated or analysed during the current study.

## Abbreviations

HF: Heart failure
NYHA: New York Heart Association
RPM: Remote patient management
TMC: Telemedical centre
TIM-HF2: The Telemedical Interventional Management in Heart Failure II
